# Osseous Sarcoidosis of the Clivus Causing Cranial Nerve VI Palsy: A Case Report

**DOI:** 10.7759/cureus.92182

**Published:** 2025-09-12

**Authors:** Xin P Wei, Taylor N LaFlam, Elham Khanafshar, Susan Kim, William D Soulsby

**Affiliations:** 1 Division of Pediatric Rheumatology, Department of Pediatrics, University of California San Francisco, San Francisco, USA; 2 Division of Cytopathology, Department of Pathology, University of California San Francisco, San Francisco, USA

**Keywords:** chronic recurrent multifocal osteomyelitis, clivus sarcoidosis, cranial nerve palsy, osseous sarcoidosis, sarcoidosis

## Abstract

Sarcoidosis is an inflammatory condition characterized by the presence of noncaseating, or nonnecrotizing, granulomas in one or more organ systems. It has a wide range of clinical presentations and often mimics other conditions, making it difficult to diagnose. Osseous sarcoidosis of the skull base is extremely rare and has not previously been reported in a young adult patient. A 20-year-old man with a prior diagnosis of chronic recurrent multifocal osteomyelitis and pulmonary nodules of unknown etiology presented with six weeks of intractable headache and eye pain, followed by acute-onset complete right-sided sixth cranial nerve palsy. Imaging revealed a lesion of his clivus, extending into the cavernous sinus, along with inflammation of his paranasal sinuses. Extensive workup was negative for infection. Histopathological analysis of a biopsy of the lesion showed chronic inflammation with nonnecrotizing granulomas without evidence of infection or vasculitis, supporting a diagnosis of sarcoidosis. He was treated with prednisone, adalimumab, and adjunctive methotrexate, and had resolution of his symptoms after six months. Osseous involvement in sarcoidosis is uncommon, particularly in the pediatric and young adult populations. Although rare, sarcoidosis should be included in the differential diagnosis for patients with sterile, inflammatory bone lesions. Careful evaluation for additional clinical features of sarcoidosis and thorough histopathological evaluation of the bone biopsy for evidence of granulomatous inflammation, as well as exclusion of alternative etiologies of granulomatous disease, help distinguish these conditions.

## Introduction

Sarcoidosis is an idiopathic inflammatory condition characterized by the presence of noncaseating, or nonnecrotizing, granulomas in one or more organ systems. While the prevalence of sarcoidosis is challenging to determine due to inconsistent and insensitive diagnostic criteria, the estimated prevalence ranges from 1 to 140 per 100,000, varying drastically across geographic regions and ethnic backgrounds [1]. The incidence of sarcoidosis peaks between the ages of 30 and 60, and it is less common in children than in adults [2].

Sarcoidosis most commonly involves the pulmonary system (up to 95% of all cases), often manifesting as reticular (fine or coarse lines on imaging) or nodular (round) opacities in the lung and bilateral hilar lymphadenopathy [3]. Other commonly affected organs include the skin (~16%), eyes (~12%), and liver (~11%), but nearly any other organ system can be affected [3]. Patients may have widely variable symptoms and disease severity, ranging from asymptomatic to complete organ failure. As a result, sarcoidosis is often misdiagnosed.

One of the less commonly affected organs involved in sarcoidosis is the bone, reported in between 3% and 13% of all cases, with the axial skeleton and the small bones of the hands and feet being the most common sites of involvement [4]. Osseous sarcoid lesions may be osteolytic (bone destruction), sclerotic (bone formation), or permeative (aggressive, poorly defined bone destruction) and are asymptomatic in many cases [5,6]. Skull involvement in sarcoidosis is rare, with fewer than two dozen cases reported [7]. Sinonasal involvement in sarcoidosis is also rare, occurring in 0.7%-6% of cases [8]. Here, we report a case of a young patient with osseous sarcoidosis of the clivus, a bone located at the base of the skull, extending into the cavernous sinus, a collection of veins within the base of the skull, causing intractable headache and a complete unilateral sixth cranial nerve (CN VI) palsy.

## Case presentation

A 20-year-old man with a previous diagnosis of chronic recurrent multifocal osteomyelitis (CRMO) presented to the emergency room with approximately six weeks of chronic, continuous headache, right-sided eye pain, and one to two weeks of worsening diplopia with rightward gaze. The patient presented to his primary care physician when his headache started. A head computed tomography (CT) revealed paranasal sinus disease, with near complete opacification of the right sphenoid sinus, obstruction of the sphenoethmoidal recesses, and mucosal thickening of the left sphenoid sinus and posterior ethmoid air cells. The patient was given a diagnosis of sinusitis. He completed a 10-day course of ampicillin/sulbactam without improvement in his symptoms. His headache continued to worsen, and he gradually developed diplopia, which prompted presentation to the emergency room.

His past medical history was significant for a diagnosis of CRMO made approximately 18 months prior. At that time, he presented with several months of severe, waxing and waning right tibial pain. Magnetic-resonance imaging (MRI) of his tibia revealed a large (14.5 cm × 1.1 cm, 270° in circumference) periosteal lesion. A biopsy was performed, which demonstrated woven bone and spindle cell proliferation with mixed inflammation without atypical cells, suggesting osteomyelitis or another inflammatory process. He was empirically treated with antibiotics without significant improvement in his symptoms, and infectious studies from the biopsy were negative. The patient received a second biopsy, which again showed reactive woven bone and mild chronic inflammation as well as rare nonnecrotizing granulomas. This second biopsy site was also sterile with negative acid-fast bacteria (AFB) and Grocott's methenamine silver stains; negative AFB, bacterial, and fungal cultures; and no pathogens detected on universal microbial DNA polymerase chain reaction (PCR). A positron-emission tomography-computed tomography (PET-CT) was performed to evaluate for systemic involvement, and the patient was found to have several hypermetabolic nodular peripheral lung consolidations. Infectious workup was negative for *Mycobacterium tuberculosis*, Histoplasma, Bartonella, Coccidioides, Toxoplasma, and HIV. Given the significant inflammatory changes on bone histology without evidence of infection, the patient was given a working diagnosis of CRMO. He was started on nonsteroidal anti-inflammatory drug (NSAID) therapy, initially naproxen, but later changed to meloxicam, with gradual resolution of his tibial pain.

On presentation to the emergency department, the patient was afebrile and found to have a complete right-sided CN VI palsy, causing diplopia from an inability to abduct his right eye, but was otherwise neurologically intact. Initial blood counts and blood culture were normal aside from elevated alkaline phosphatase (214 U/L) (Table 1). Inflammatory markers were elevated with an erythrocyte sedimentation rate of 47 mm/hour and a C-reactive protein of 18 mg/L. Lumbar puncture had normal opening pressure, and cerebrospinal fluid showed mild pleocytosis (10 white blood cells/mm^3^), normal glucose, mildly elevated protein, and negative bacterial culture. The ophthalmologic exam was normal, aside from the previously noted ophthalmoplegia.

**Table 1:** Laboratory results CU: chemiluminescence units; RPR: rapid plasma regain; PCR: polymerase chain reaction; IgG: immunoglobulin G

Category	Laboratory test	Result	Reference
Basic labs	Alkaline phosphatase	214 U/L	36-130 U/L
	Erythrocyte sedimentation rate	47 mm/hour	2-28 mm/hour
	C-reactive protein	18 mg/L	<5.1 mg/L
	White blood cell	8.2 × 10^9^/L	4.5-13.2 × 10^9^/L
	Hemoglobin	14.4 g/dL	13.6-17.5 g/dL
	Platelet	246 × 10^9^/L	140-450 × 10^9^/L
Infectious workup	QuantiFERON-TB gold	Negative	Negative
	Coccidioides Ab complement fixation	<1:2	<1:2
	Coccidioides Ab	Negative	Negative
	Cryptococcus Ag	Negative	Negative
	Aspergillus galactomannan Ag index	0.032	<0.5
	*Treponema pallidum* (treponemal)	Nonreactive	Nonreactive
	*Treponema pallidum* (RPR)	Nonreactive	Nonreactive
	Lyme Ab	Negative	Negative
	COVID-19 PCR	Not detected	Not detected
	Hepatitis B (HBsAb, HBcAb, HBsAg)	Negative	Negative
	Strongyloides IgG Ab	Negative	Negative
Miscellaneous	Anti-proteinase 3 Ab	<10 CU	<20 CU
	Anti-myeloperoxidase Ab	<10 CU	<20 CU
	Angiotensin-converting enzyme	22 U/L	9-67 U/L
	Lysozyme	3.8 mcg/mL	5-11 mcg/mL
	1,25-dihydroxyvitamin D	59 pg/mL	20-79 pg/mL
	Calcium	9.5 mg/dL	8.6-10.3 mg/dL
	Neutrophil oxidative index	83	≥22
	Blood culture	Negative	Negative
Cerebrospinal fluid	Opening pressure	20 cmH_2_O	6-25 cmH_2_O
	White blood cell	10 × 10^6^/L	0-10 × 10^6^/L
	Glucose	56 mg/dL	40-70 mg/dL
	Protein	52 mg/dL	15-50 mg/dL
	Bacterial culture	Negative	Negative

**Figure 1:**
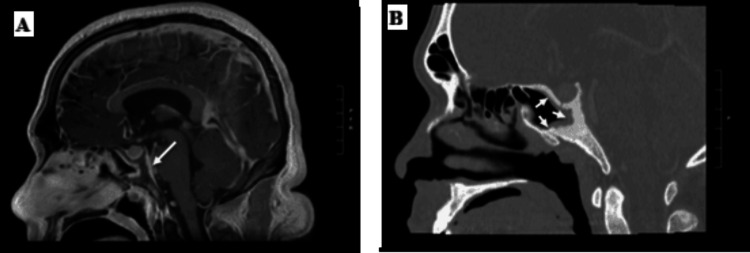
MRI and CT imaging of the clivus and surrounding sinuses (A) MRI sagittal T1 sequence with intravenous contrast shows abnormal marrow signal and enhancement of the clivus and dorsum sella (arrow). (B) CT of sinuses, sagittal view with bone window, shows thickening of the walls of the sphenoid sinuses (arrows) MRI: magnetic resonance imaging; CT: computed tomography

The patient underwent MRI of his brain, which showed abnormal marrow signal and enhancement of the clivus and dorsum sella extending into the cavernous sinus, which was concerning for an infectious or inflammatory osteomyelitis (Figure 1A). Mucosal enhancement was seen along the posterior aspect of the sphenoid sinus, and there was mucosal thickening of the paranasal sinuses. On CT imaging of his sinuses, the patient was found to have increased sclerosis and osseous thickening of the dorsal and posterior walls of the sphenoid sinuses, extending into the clivus (Figure 1B). On PET-CT, hypermetabolism and cortical thickening of the clivus, as well as a new nodular consolidation in the right lung apex, were observed.

A broad infectious and rheumatologic workup was performed. Serological evaluation was negative for *M. tuberculosis* (QuantiFERON-TB Gold), Coccidioides (antibody, Ab, and complement fixation), Cryptococcus antigen (Ag), Aspergillus (Galactomannan Ag), *Treponema pallidum* (rapid plasma reagin, treponemal), Lyme (Ab), COVID-19 (PCR), Hepatitis B (Hepatitis B surface Ab, Hepatitis B core Ab, Hepatitis B surface Ag), and Strongyloides (IgG Ab). Neutrophil oxidative index was normal. Serum metagenomics next-generation sequencing returned *Acinetobacter haemolyticus* and *Brevibacterium casei*. However, in consultation with infectious disease, these organisms were concluded to be contaminant organisms as they have previously only been observed in osteomyelitis in the setting of orthopedic procedures. The patient was negative for anti-proteinase-3 and anti-myeloperoxidase antibodies. He was found to have normal levels of angiotensin-converting enzyme (ACE) (22 U/L), lysozyme (3.8 mcg/mL), 1,25-dihydroxyvitamin D (59 pg/mL), and calcium (9.5 mg/dL). Reevaluation of his prior tibial biopsy did not show evidence of Langerhans cell histiocytosis or Rosai-Dorfman disease.

**Figure 2:**
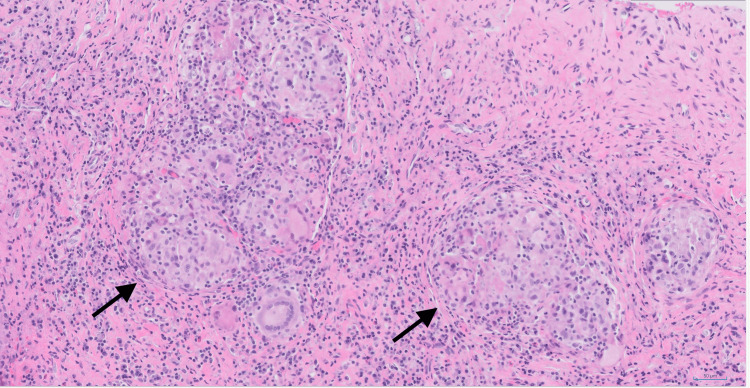
Clivus biopsy histopathology Well-formed nonnecrotizing granulomas (black arrows) with surrounding inflammation (hematoxylin and eosin stain, 200×)

Biopsies of the sphenoid sinus, the mucosa in the clival area, and the clival bone were obtained. Histologic sections showed sinonasal respiratory mucosa with well-formed, nonnecrotizing granulomas in a background of mixed acute and chronic inflammation with reactive changes in the bone (Figure 2). Lamellated calcifications were found in a subset of the granulomas. No histopathologic specific evidence of infection was seen, and the accompanying universal PCR was also negative. The vasculature was intact without significant inflammation or necrosis. CD1a staining did not show evidence of Langerhans cell histiocytosis. IgG4 staining was performed, and the histologic appearance was not consistent with IgG4-related disease (IgG4-RD). Finally, there was no evidence of a neoplastic process. These biopsy results, which demonstrated nonnecrotizing granulomatous inflammation, consistent with his prior bone biopsy and coupled with the presence of pulmonary nodules and absence of markers for other diseases, marked a turning point in our diagnostic process, strongly suggesting sarcoidosis as the unifying diagnosis.

**Figure 3:**
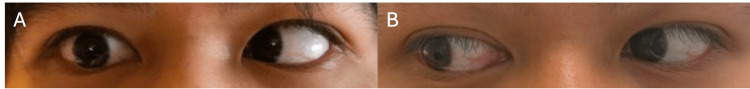
CN VI palsy and resolution following treatment (A) CN VI palsy of the right eye during admission, before starting treatment. (B) One year after starting treatment CN VI: sixth cranial nerve

During the admission, the patient's headache and ocular pain improved with naproxen, although he had persistent right CN VI palsy (Figure 3A). After being diagnosed with sarcoidosis, the patient was started on prednisone 60 mg daily, adalimumab 40 mg subcutaneous every 14 days, and low-dose methotrexate, 10 mg *per os* (PO) every seven days, with the intent of reducing the risk of developing anti-adalimumab neutralizing antibodies. Over the course of several months, the patient had a gradual resolution of his CN VI palsy (Figure 3B). The patient tolerated a prednisone wean over the course of four months to 5 mg daily and continued adalimumab and methotrexate. Follow-up chest CT at three months showed near resolution of previously noted right apical nodules and no additional nodules.

Approximately 18 months following discharge, the patient reported having progressive worsening positional headaches and episodic tunnel vision. He was found to have bitemporal hemianopsia without diplopia on vision exam. MRI brain showed abnormal enhancement of the pituitary gland extending into the optic chiasm. To confirm that these symptoms were a result of the progression of his sarcoidosis, a pituitary gland biopsy was performed. The resulting pathology showed well-formed nonnecrotizing granulomas with multinucleated giant cells in a background of lymphocytic inflammatory infiltrates (hypophysitis), confirming a diagnosis of neurosarcoidosis. The patient was treated with five days of 1 g intravenous methylprednisolone, followed by a prednisone taper starting at 80 mg daily. He was also transitioned from adalimumab to infliximab 5 mg/kg monthly, and his methotrexate was increased to 15 mg PO weekly. His visual defects have since completely resolved.

## Discussion

Here, we report a case of osseous sarcoidosis of the skull base leading to cranial nerve VI palsy and headache. Sarcoidosis is a challenging diagnosis. It lacks definitive diagnostic criteria and requires exclusion of other similar conditions. Only two prior pediatric cases of skull involvement in sarcoidosis have been reported, both affecting the cranial convexity [9,10]. To our knowledge, this is the first reported case of osseous sarcoid of the skull base in a young adult patient.

In this case, the patient's presentation was initially concerning for a primary neurological disease/disorder. However, imaging helped localize the main lesion to the clivus, causing inflammation of nearby structures, including CN VI. For this patient who also had concurrent chronic sinusitis and nodular peripheral lung consolidations of unclear etiology, infection was initially high on the differential, though ruled out after extensive workup. Given sinus and pulmonary disease in the setting of systemic inflammation, antineutrophil cytoplasmic antibody-associated vasculitis was also considered but excluded by serologies and biopsy. While IgG4-RD was considered, this patient did not have any common manifestations of IgG4-RD, such as retroperitoneal lymphadenopathy or lacrimal gland infiltration. Furthermore, he had normal serum levels of IgG4, and the proportion of IgG4 plasma cells on his biopsy did not reach typical thresholds for IgG4-RD [11]. Given the lack of systemic manifestations, a malignant etiology, such as lymphoma, was thought to be less likely, and the biopsy confirmed the absence of a neoplastic process.

Ultimately, the pathological finding of well-formed, nonnecrotizing granulomas in the clival biopsy (as well as in the prior tibial biopsy) and the absence of findings supportive of other conditions represented the key inflection point in our diagnostic thinking and made sarcoidosis the most likely diagnosis (differential diagnosis of pediatric sarcoidosis listed in Table 2) [12]. The diagnosis of sarcoidosis also provided a unifying explanation for his osseous and pulmonary nodules. Although he lacked hilar lymphadenopathy, pulmonary infiltrates or nodules can be seen in 10%-15% of sarcoidosis patients [13]. Furthermore, although this patient did not have elevated serum ACE, lysozyme, or 1,25-dihydroxyvitamin D levels, it is important to note that the sensitivity of these markers is below 80% [14]. Cranial nerve palsy is also a rare complication of sarcoidosis. In this case, the direct osseous sarcoidosis involvement of the clivus and ipsilateral cavernous sinus surrounding the CN VI favors an osseous origin of this patient's cranial nerve deficit. However, it is difficult to rule out direct involvement of the cranial nerve itself. In summary, the patient's tibial lesion, chronic inflammation of his paranasal sinuses, granulomatous skull base lesion, and pulmonary nodules are compatible with sarcoidosis, which is further confirmed by nonnecrotizing granulomatous inflammation of his pituitary gland during the subsequent progression of his disease.

**Table 2:** Differential diagnosis for pediatric sarcoidosis

Category	Differential diagnosis
Chronic granulomatous infections	Mycobacterial infections
	Fungal infections
Other rheumatologic disorders	Granulomatosis with polyangiitis
	Eosinophilic granulomatosis with polyangiitis
	Microscopic polyangiitis
	Juvenile idiopathic arthritis
	Idiopathic inflammatory uveitis
	IgG4-related disease
	Chronic recurrent multifocal osteomyelitis/chronic nonbacterial osteitis
Other systemic autoimmune disorders	Crohn’s disease
Primary immunodeficiencies	Chronic granulomatous disease
	Common variable immunodeficiency
Malignancies	Lymphoma (Hodgkin and Non-Hodgkin)
	Leukemia
	Langerhans cell histiocytosis

Of note, this patient had an initial diagnosis of CRMO after two tibial biopsies showed sterile inflammatory changes. In retrospect, this was likely a misdiagnosis, and the patient instead had an atypical presentation of sarcoidosis on initial presentation, which we did not fully appreciate until his second presentation and clival biopsy. CRMO is a rare pediatric condition characterized by insidious onset of bone pain, lytic and sclerotic bone lesions on imaging, and a sterile inflammation of bone on biopsy [15]. Granulomas have been seen in CRMO and can also occur as reactive changes at prior biopsy sites, which contributed to the incorporation of this pathologic finding into his initial diagnosis [16]. This patient's tibial symptoms improved with NSAIDs, but it is unclear whether this may represent successful treatment for a distinct diagnosis of CRMO or spontaneous remission of sarcoidosis, which can occur in up to 60%-70% of patients (the rate of spontaneous remission specifically for osseous manifestations of sarcoidosis is unclear) [17]. This case reveals the underappreciated overlap between these two conditions and highlights the challenges of diagnosing CRMO and osseous sarcoidosis in patients without other, more typical features of sarcoidosis.

The mainstay of treatment for sarcoidosis is corticosteroids, with methotrexate often used as a second-line agent, based on limited observational data and expert opinion. Randomized controlled trials (RCTs) assessing and comparing their effectiveness are ongoing [18]. Tumor necrosis factor inhibitors have often been used off-label for the treatment of sarcoidosis, particularly in steroid-refractory cases. However, their efficacy in retrospective studies and limited RCTs for pulmonary and extrapulmonary sarcoidosis has been mixed [19,20]. Clinically and radiologically, our patient initially responded to a combination therapy of prednisone, adalimumab, and adjunctive methotrexate. He later developed neurosarcoidosis of his pituitary gland on this regimen, requiring pulse steroids for the acute management of his symptoms. For now, he remains stable on a regimen of prednisone, infliximab, and methotrexate.

## Conclusions

Although rare, sarcoidosis should be considered in patients with cranial nerve deficits or sterile osseous inflammation, including of the skull base. In this case, multiple biopsies showing nonnecrotizing granulomas at different sites were essential in distinguishing sarcoidosis from mimics such as CRMO and infection. The unusual location of involvement and initial misleading presentation underscore the need for a high index of suspicion and reexamination of earlier diagnoses when patients develop new organ involvement that points toward a systemic granulomatous disease.
